# Identification and experimental validation of prognostic genes related to cytochrome c in breast cancer

**DOI:** 10.3389/fgene.2025.1627134

**Published:** 2025-08-11

**Authors:** Huimin Yu, Shihong Li, Jian Wu, Haobin Wang

**Affiliations:** ^1^ College of Medicine, Southwest Jiaotong University, Chengdu, China; ^2^ General Hospital of Western Theater Command, Chengdu, China; ^3^ Department of Thyroid and Breast Surgery, The Third People’s Hospital of Chengdu, Chengdu, China; ^4^ Department of Thyroid and Breast Surgery, The Sixth People’s Hospital of Chengdu, Chengdu, China

**Keywords:** breast cancer, cytochrome c, prognostic genes, risk model, cytokine-cytokine receptor interaction

## Abstract

Breast cancer (BC) is one of the most prevalent malignant diseases affecting women. Cytochrome c (Cyt c) plays a critical role in various pathological processes, however, its precise mechanism in BC remains unclear. This study aimed to identify prognostic genes linked to Cyt c in BC and explore their underlying mechanisms. Transcriptome data related to BC were initially obtained from TCGA and GEO database. Prognostic genes were identified through differential expression analysis, univariate Cox regression, and LASSO analysis. A risk model was subsequently developed and validated. Additionally, enrichment analysis, immune microenvironment analysis, and the construction of a TFs-mRNA network were conducted. Finally, the expression levels of prognostic genes were examined in both tumor and normal tissue samples, with confirmation through RT-qPCR. Eight prognostic genes (*CETP*, *CLEC11A*, *CYP2A6*, *CYP2A7*, *GZMB*, *HGF*, *LDHC*, and *PLAU*) were identified. The risk model demonstrated that low-risk individuals have significantly higher survival rates. GSEA results indicated that seven of the prognostic genes are notably enriched in the “cytokine-cytokine receptor interaction” pathway. Transcription factors, such as ATF3 and RUNX1, were found to regulate these prognostic genes. Furthermore, immune cell profiles revealed significant differences between high-risk and low-risk groups. Bioinformatics and RT-qPCR analyses confirmed that *CETP* and *HGF* are upregulated in normal tissues, while *CLEC11A* and *PLAU* showed higher expression in BC tissues. This study identified eight Cyt c-related prognostic genes and developed a risk model, offering new insights into personalized treatment and prognosis for BC.

## 1 Introduction

Breast cancer (BC) is among the most prevalent cancers globally and the leading cause of cancer-related mortality in women, with an estimated 2.3 million new cases annually. By 2030, this number is expected to rise to 2.7 million new cases per year, accompanied by 870,000 deaths (Sung et al., 2021). BC classification is based on histological features or the expression of biomarkers such as estrogen receptor (ER), progesterone receptor (PR), and human epidermal growth factor receptor 2 (HER2). Additional biomarkers, including Ki-67, Mib1, E-Cadherin, and P53, further refine classification (Łukasiewicz et al., 2021). Treatment and prognosis are closely linked to BC stage and classification. Despite the existence of molecular markers like MAST1, PRDM14, and ZNF177 that predict prognosis (Mao et al., 2021), precise prognostic evaluation remains challenging, necessitating the identification of novel BC-related prognostic genes. Such discoveries are essential for improving patient outcomes and guiding timely, targeted therapies.

Cytochrome c (Cyt c) is a promising pro-apoptotic protein in cancer therapy. As a heme-containing metalloprotein, Cyt c functions as an electron transfer protein within the mitochondrial respiratory chain, supporting healthy cell proliferation. In the cytoplasm, it triggers intrinsic apoptosis to eliminate damaged cells (Delinois et al., 2021). In drug-induced BC cell death, NAT1 deficiency shifts the death pathway from Cyt c-dependent apoptosis to necroptosis, diminishing the response to cytotoxic treatments (McAleese et al., 2022). Retinoic acid and retinol regulate Cyt c’s oxidative phosphorylation (OXPHOS), apoptosis, and redox balance in BC cells, contributing to mitochondrial energy homeostasis (Surmacki and Abramczyk, 2023). In European populations, Cyt c oxidase subunit 8A and ADP-ribose pyrophosphatase serve as protective factors against BC (Miao et al., 2024). Conversely, Cyt c oxidase assembly factor (COA6), upregulated in BC, correlates with poor survival outcomes and may facilitate BC progression via OXPHOS regulation (Jin et al., 2024). Studies have shown that breast cancer cells show unusual sensitivity to Cyt c-induced apoptosis compared to normal breast cells. This sensitivity is due to enhanced recruitment of caspase-9 to the Apaf-1 caspase recruitment domain. Enhanced caspase activation is mediated by PHAPI, which is also overexpressed in breast cancer (Schafer et al., 2006). LRG1 has been shown to compete with the apoptosis activator Apaf-1 for binding to Cyt c. In the breast cancer cell line MCF-7, LRG1 can prevent apoptosis. Cyt c is the only protein that can be co-precipitated with LRG1. The increase in intracellular LRG1 levels increases the cytoplasmic concentration of Cyt c, which is required for the induction of apoptosis, thereby inhibiting the occurrence of the intrinsic apoptotic pathway of the cell, and this process is independent of apoptotic signals (Jemmerson et al., 2021). While connections between Cyt c and BC exist, its precise molecular mechanisms in cancer development remain unclear.

In this study, bioinformatics analyses of publicly available transcriptome data were performed to identify prognostic genes linked to Cyt c in BC. Additionally, risk and nomogram models based on these genes were constructed, followed by enrichment, immune microenvironment, and drug sensitivity analyses. This research offers novel insights into BC diagnosis, prognosis, and treatment.

## 2 Materials and methods

### 2.1 Source of data

This study utilized the TCGA-BRCA dataset (retrieved on 23 July 2024) from The Cancer Genome Atlas (TCGA, https://portal.gdc.cancer.gov/), which provides comprehensive information on patient age, sex, tumor grade, and stage. The dataset included 1,096 tumor tissue samples from patients with BC, all with survival data, and 113 normal tissue samples. Additionally, BC-related datasets GSE20685 (GPL570) and GSE42568 (GPL570) were retrieved from the Gene Expression Omnibus (GEO, http://www.ncbi.nlm.nih.gov/geo/). Both datasets were of the microarray data type. The GSE20685 dataset comprised 327 tumor tissue specimens from patients with BC, all with survival data. The GSE42568 dataset contained 104 tumor tissue specimens and 17 normal tissue specimens from patients with BC. Furthermore, 781 Cyt c-related genes (CCRGs) were gathered from the literature (Tang et al., 2022) (Supplementary Table S1).

### 2.2 Identification of differentially expressed genes (DEGs)

To identify DEGs between tumor and normal tissue samples, the “DESeq2” package (v 1.38.0) (Love et al., 2014) was employed for analysis within the TCGA-BRCA dataset (|log_2_ fold change (FC)| >0.5, adj. P < 0.05). The “ggplot2” package (v 3.3.6) (Gustavsson et al., 2022) was used to generate a volcano plot, and the “pheatmap” package (v 1.0.12) (Zhang X. et al., 2023) was utilized to create a heatmap illustrating the top 10 upregulated and downregulated genes, ranked by |log_2_FC| values.

### 2.3 Identification and enrichment assessment of candidate genes

To identify candidate genes associated with Cyt c in BC, the “VennDiagram” package (v 1.7.3) (Chen and Boutros, 2011) was used to intersect the DEGs with the CCRGs, thus obtaining the candidate genes. Gene Ontology (GO) and Kyoto Encyclopedia of Genes and Genomes (KEGG) enrichment analyses were performed on the candidate genes using the “clusterProfiler” package (v 4.6.2) (Wu et al., 2021) to explore their biological functions and pathways (P < 0.05). The top 5 entries from each GO category (biological processes [BP], cellular components [CC], and molecular functions [MF]) and the top 10 KEGG pathways were presented according to their significance.

### 2.4 Identification of prognostic genes

In the TCGA-BRCA dataset, univariate Cox regression analysis of the candidate genes was conducted using the “survival” package (v 3.5-3) (Liu et al., 2021), with criteria set for a hazard ratio (HR) not equal to 1, P < 0.05, and a P-value of the proportional hazards (PH) assumption test greater than 0.05. Genes passing both the univariate Cox analysis and PH test were considered candidate prognostic genes. Subsequently, Least Absolute Shrinkage and Selection Operator (LASSO) analysis was performed on these genes using the “glmnet” package (v 4.1-4) (Friedman et al., 2010), with the most appropriate lambda value for prognostic gene selection, and the minimum criterion for the best lambda value was determined by 10-fold cross validation.

### 2.5 Construction and validation of the risk model

The risk score for each patient in the TCGA-BRCA dataset was then calculated using the following equation:

$$\text{Risk score}=\sum_{i=1}^{n}\text{coef}\left({\text{gene}}_{i}\right)*\text{expr}\left({\text{gene}}_{i}\right)$$

In this equation, Coef represents the risk coefficient attributed to individual genes, while expr denotes the expression of individual genes. In the TCGA-BRCA dataset, patients with overall survival (OS) data were stratified into high-risk group (HRG) and low-risk group (LRG) based on the optimal cutoff value of risk score. Kaplan-Meier (K-M) survival analysis was performed using the “survival” package (v 3.5-3) (Liu et al., 2021), and the resulting K-M curves were generated (P < 0.05). Receiver operating characteristic (ROC) analysis was conducted with the “survivalROC” package (v 1.0.3.1) (Zhang S. et al., 2023), producing ROC curves for 1, 3, and 5-year intervals, with the area under the curve (AUC) calculated (AUC >0.6). Additionally, the expression levels of prognostic genes in the HRG and LRG were analyzed and visualized using the “pheatmap” package (v 1.0.12) (Zhang X. et al., 2023). The robustness and reliability of the risk model were further validated using the GSE20685 dataset.

### 2.6 Construction and validation of the nomogram

In the BC samples from the TCGA-BRCA dataset, univariate Cox analysis (P < 0.05, HR ≠ 1) and the PH test (P > 0.05) were conducted on risk score, N stage, age, gender, T stage, M stage, and stage using the “survival” package (v 3.5-3) (Liu et al., 2021). Following these analyses, multivariate Cox regression (with HR ≠ 1 and P < 0.05) was performed to identify independent prognostic factors among variables meeting the criteria. A nomogram model was then constructed using the “rms” package (v 6.5-0) (Liu et al., 2021) to predict the 1, 3, and 5-year survival probabilities of patients with BC based on these independent prognostic factors. The predictive performance of the nomogram was evaluated using the “survivalROC” package (v 1.0.3.1) (Zhang S. et al., 2023) through ROC analysis, with the AUC calculated (AUC >0.6). In addition, the performance of the prognostic model was robustly assessed by performing 100 self-service samples (Bootstrap) of the original dataset with playback, constructing a Cox proportional risk regression model on each sample, and calculating the 1-, 3-, and 5-year time points and the corresponding AUC values based on the model’s linear predictive values.

### 2.7 Correlation analysis of clinical features

In the TCGA-BRCA dataset, patients with BC were classified into distinct clinical subgroups according to various clinical characteristics. The Wilcoxon test was used to compare differences in risk scores across these subgroups, and visual representations were generated using the “ggplot2” package (v 3.3.6) (Gustavsson et al., 2022).

### 2.8 Gene-set variation analysis (GSVA) and gene-set enrichment analysis (GSEA)

To investigate the changes in KEGG pathways between the high-risk and low-risk groups in the TCGA-BRCA dataset, the gene set “c2.all.v2023.2.Hs.symbols” was retrieved from MSigDB (https://www.gsea-msigdb.org/gsea/msigdb/index.jsp) as the background. GSVA was performed using the “GSVA” package (v 1.46.0) (Hänzelmann et al., 2013) to obtain enrichment scores for different pathways. Pathways enriched in each risk group were then analyzed and compared using the “limma” package (v 3.54.0) (Ritchie et al., 2015). Significant pathways were identified based on a P-value threshold of <0.05. Additionally, the “cor” function in R was used to compute Spearman correlations between prognostic genes and other genes in the TCGA-BRCA dataset, with results sorted by correlation strength. The C2:KEGG gene sets were used as the background for this analysis. Finally, GSEA was performed with the “clusterProfiler” package (v 4.6.2) (Wu et al., 2021), using criteria of |False Discovery Rate (FDR)| < 0.25, |Normalized Enrichment Score (NES)| > 1, and P < 0.05, with the top 5 most significant pathways displayed. Finally, the signaling pathways were visualized using the R package “enrichplot” (1.18.0) (Yu et al., 2015) according to p.adjust <0.05, and filtered to show the top 5 signaling pathways enriched for each prognostic gene.

### 2.9 Immune microenvironment analysis

The immune microenvironment characteristics of two risk groups in the TCGA-BRCA dataset were analyzed using the CIBERSORT algorithm to determine the relative abundances of 22 distinct immune cell types (Chen et al., 2018). The Wilcoxon test was then applied to assess the infiltration differences of these immune cell types between HRG and LRG, with immune cells exhibiting significant differences (P < 0.05) identified. Results were visualized through box plots created using the “ggplot2” package (v 3.3.6) (Gustavsson et al., 2022). Next, the “cor” function in the R package was used to explore Spearman correlations among differentially expressed immune cells, as well as between these cells and prognostic genes and risk scores (|correlation (cor)| > 0.3, P < 0.05).

To investigate the potential clinical efficacy of immunotherapy in HRG and LRG, the “ESTIMATE” package (v 1.0.13) (Chakraborty et al., 2018) was employed to calculate the ESTIMATE score, immune score, and stromal score in BC samples. The Wilcoxon test was further used to evaluate differences in ESTIMATE score, stromal score, immune score, and immune checkpoints (Wang et al., 2022) between HRG and LRG patients (P < 0.05).

### 2.10 Gene mutation analysis

To investigate the genetic mutation between high and low risk groups in BC patients. The R package “TCGAmutations (v 0.3.0)” (Huang et al., 2023) was used to download the TCGA_BRCA somatic mutation information (mutation data was obtained from Whole Exome Sequencing, WES). Samples from High risk and Low risk groups were selected, and BC samples were analyzed for somatic mutations based on subgroups. In TCGA-BRCA dataset, “MAfTools” package (v 2.14.0) (Mayakonda et al., 2018) was then employed to depict the top 20 mutated genes in HRG and LRG. Meanwhile, “MAfTools” package (v 2.14.0) (Mayakonda et al., 2018) was also employed to calculate the tumor mutation burden (TMB) of HRG and LRG. In TMB analysis, log2 (TMB+1) transformation was used to eliminate skewed distribution. The Wilcoxon test was deployed to examine differences in TMB within HRG and LRG (P < 0.05). Finally, based on the BC samples in TCGA-BRCA dataset, “MAfTools” package (v 2.14.0) (Mayakonda et al., 2018) was also adopted to calculate the mutation frequency of prognostic genes in accordance with formula: the ratio of mutated samples to cancer samples.

### 2.11 Drug sensitivity analysis and construction of the TFs-mRNAs network

For drug sensitivity prediction, the “oncoPredict” package (v 1.2) (Maeser et al., 2021) was utilized to estimate the half-maximal inhibitory concentration (IC_50_) values for 198 drugs from the GDSC database (https://www.cancerrxgene.org/) in BC samples from the TCGA-BRCA dataset. The Wilcoxon test was used to compare IC_50_ values between HRG and LRG groups (P < 0.05). Furthermore, the “psych” package (v 2.2.9) (Kyriazos and Poga-Kyriazou, 2023) was applied to evaluate correlations between the risk score and the top 10 drugs with the largest IC_50_ differences between the two risk groups (|cor| > 0.3, P < 0.05).

To identify transcription factors (TFs) regulating prognostic genes, the KnockTF database (http://www.licpathway.net/KnockTF/index.html) was consulted. A TF-mRNA regulatory network was constructed using Cytoscape software (v 3.10.2) (Franz et al., 2023).

### 2.12 Content analysis of prognostic genes and reverse transcription-quantitative polymerase chain reaction (RT-qPCR) validation

The Wilcoxon test was employed to assess the differences in the protein content of prognostic genes between BC and normal samples (P < 0.05). This analysis was further validated using the GSE42568 dataset.

To further validate the expression of prognostic genes in BC tissues compared to normal tissues, RT-qPCR assays were performed. Five tumor tissue samples from patients with BC and five adjacent non-tumor tissue samples were collected from Chengdu Sixth People’s Hospital for this purpose. The study was approved by the Ethics Committee of Chengdu Sixth People’s Hospital, and informed consent was obtained from all patients.

Total RNA was extracted from the samples using TRIZOL (Ambion, Austin, USA) following the manufacturer’s instructions. The first strand of complementary DNA (cDNA) was synthesized from 2 μg of total RNA using the SweScript first-strand cDNA synthesis kit (Servicebio, Wuhan, China), according to the manufacturer’s protocol. RT-qPCR was conducted using the 2xUniversal Blue SYBR Green qPCR Master Mix supplied by Servicebio. Detailed primer sequences and reaction procedures are provided in Supplementary Table S2. GAPDH was used as an internal control to ensure the stability and reliability of the experimental results. Gene expression was quantified using the 2^−ΔΔCT^ method (Livak and Schmittgen, 2001), and results were visualized using GraphPad Prism 10.

### 2.13 Statistical analysis

Statistical evaluations were conducted utilizing R software (v 4.2.2), and the Wilcoxon test was deployed to determine significant differences among groups. In univariate Cox regression analysis with HR ≠ 1 & p < 0.05, PH hypothesis test p > 0.05 as screening condition. The p.adjust function in the R language stats package was used to correct the false discovery rate (False Discovery Rate, FDR) of the p-value obtained from a single factor using the Benjamini-Hochberg (BH) method. The significance threshold was set to q < 0.05 to ensure that the false discovery rate remained low under multiple tests. In Lasso regression analysis, the optimal lambda value was used for prognostic gene screening. In GSVA analysis, p < 0.05. In GSEA analysis, |False Discovery Rate (FDR)| < 0.25, |Normalized Enrichment Score (NES)| > 1, and P < 0.05. GSEA was performed using the GSEA function in the clusterProfiler package, and the p-values in the results were corrected for multiple hypothesis testing. In immune microenvironment analysis, the raw p-values were first obtained by performing a nonparametric Wilcoxon rank sum test for each immune cell type and 10 immune checkpoint genes between the two groups using the wilcox_test function in the rstatix package. Subsequently, the Benjamini-Hochberg (BH) method was used to correct the multiple tests for false discovery rate (FDR) using the adjust_pvalue function in the rstatix package to obtain the corrected p-value (p.adj). In gene mutation analysis, Benjamini-Hochberg method was used for multiple hypothesis correction. In RT-qPCR, the Ct values were compared using paired, two-tailed t-tests, which were computed utilizing GraphPad Prism software. A p-value below 0.05 was considered to denote statistical significance.

## 3 Results

### 3.1 A total of 171 candidate genes were identified, and enrichment analyses were carried out

**FIGURE 1:**
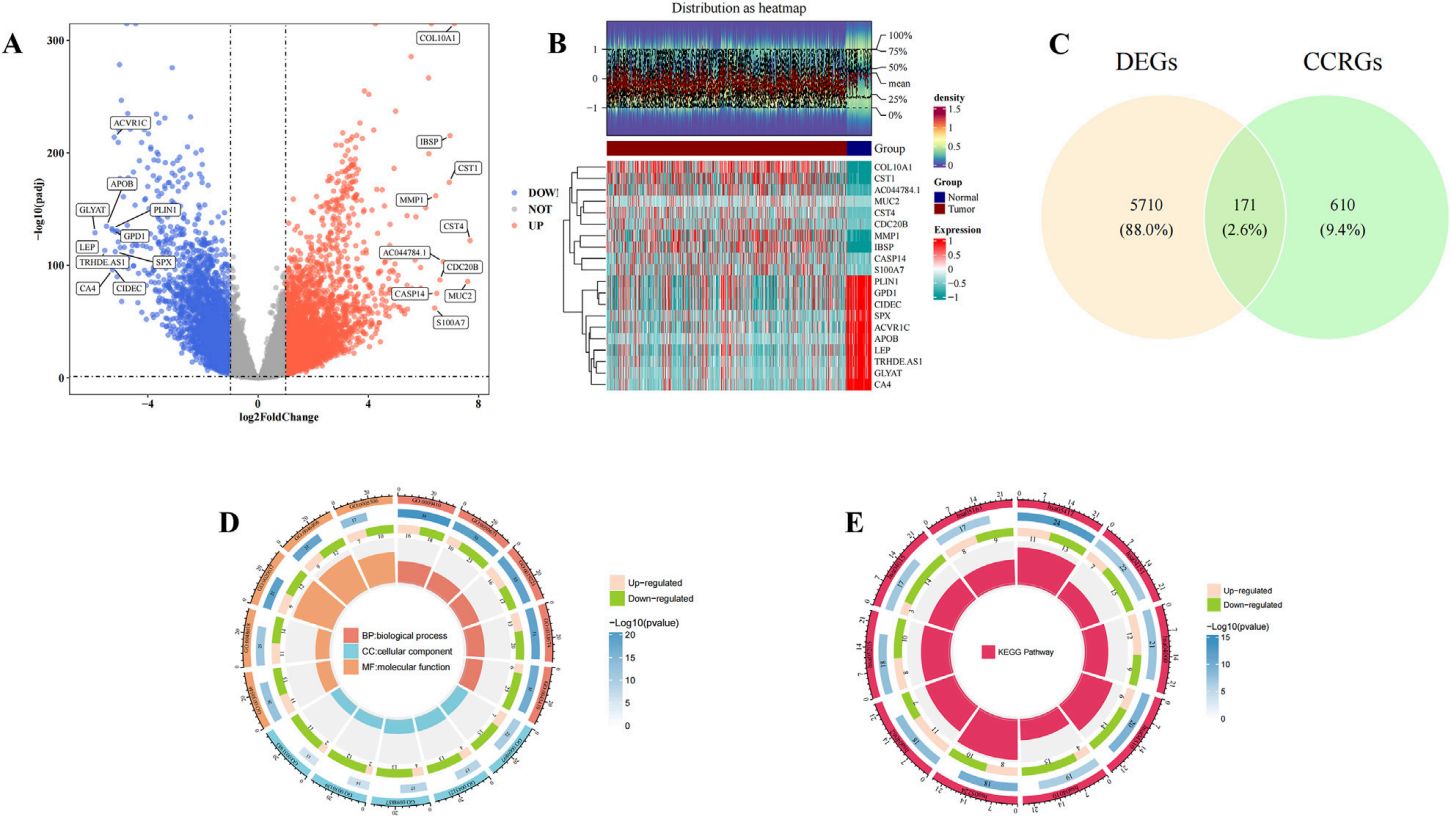
Candidate gene selection and enrichment analysis. **(A)** Volcano plots showed upregulated and downregulated genes in normal patients and Breast cancer (BC) patients in the The Cancer Genome Atlas - Breast Invasive Carcinoma (TCGA-BRCA) dataset. Horizontal coordinates indicate multiplicity of differences (tumor/normal, logarithmic), vertical coordinates indicate -log10(padj), each dot in the volcano plot represents a gene, and the color of the dots-blue indicates downregulation of gene expression, red indicates upregulation of gene expression. **(B)** Heat map showed upregulated and downregulated genes in normal patients and Breast cancer (BC) patients in the The Cancer Genome Atlas - Breast Invasive Carcinoma (TCGA-BRCA) dataset. The top heatmap from blue to red indicates the increase in the number of samples, the middle indicates the grouping of samples (Tumor group and Normal group); the bottom heatmap, each row indicates the expression profile of each gene in different samples, and each column indicates the expression profile of all DEGs in each sample. The dendrogram on the left side represents the results of cluster analysis of different genes from different samples. Each small square in the heatmap on the right side represents each gene, and its color represents the magnitude of gene expression, the darker the color indicates the higher expression of the gene (red is high expression, blue is low expression). **(C)** Venn diagram showed the screening of 171 candidate genes. Yellow indicates differential genes and green indicates cytochrome C-related genes. **(D,E)** Gene Ontology (GO) and Kyoto Encyclopedia of Genes and Genomes (KEGG) enrichment analysis. In the GO circle diagram, clockwise rotation is the horizontal coordinate, red, blue and yellow stand for BP, CC and MF, respectively, and the extension from the center to the circumference of the circle is the vertical coordinate, with a total of four levels, the first being the GO function, the second being the number of up- and downregulated genes, the third being the total number of enriched genes, and the fourth being the GO number. In the KEGG circle diagram, the clockwise rotation is the horizontal coordinate, the red color represents the KEGG pathway, and the extension from the center to the circumference of the circle is the vertical coordinate, which has four levels in total: the first level is the KEGG pathway, the second is the number of up- and downregulated genes, the third is the total number of enriched genes, and the fourth is the KEGG number.

A total of 5,881 DEGs were identified between normal and BC samples in the TCGA-BRCA dataset. Among these, 3,475 genes were upregulated, while 2,406 genes were downregulated in BC samples (Figures 1A,B). A set of 171 candidate genes was derived from the intersection of these DEGs and 781 CCRGs (Figure 1C). These candidate genes were significantly enriched in 2,336 biological process (BP) terms, including response to xenobiotic stimulus and cytokine-mediated signaling pathway, 94 cellular component (CC) terms such as membrane raft and membrane microdomain, and 187 molecular function (MF) terms such as cytokine activity and receptor-ligand activity (P < 0.05) (Figure 1D; Supplementary Table S3). Additionally, these genes were concentrated in 146 KEGG pathways, notably the PI3K-Akt signaling pathway (P < 0.05) (Figure 1E; Supplementary Table S4). These results suggest that candidate genes may play a role in responding to foreign stimuli and cell signaling, which may help us understand the biological mechanisms involved.

### 3.2 A total of eight prognostic genes were identified, and risk models were constructed

**FIGURE 2:**
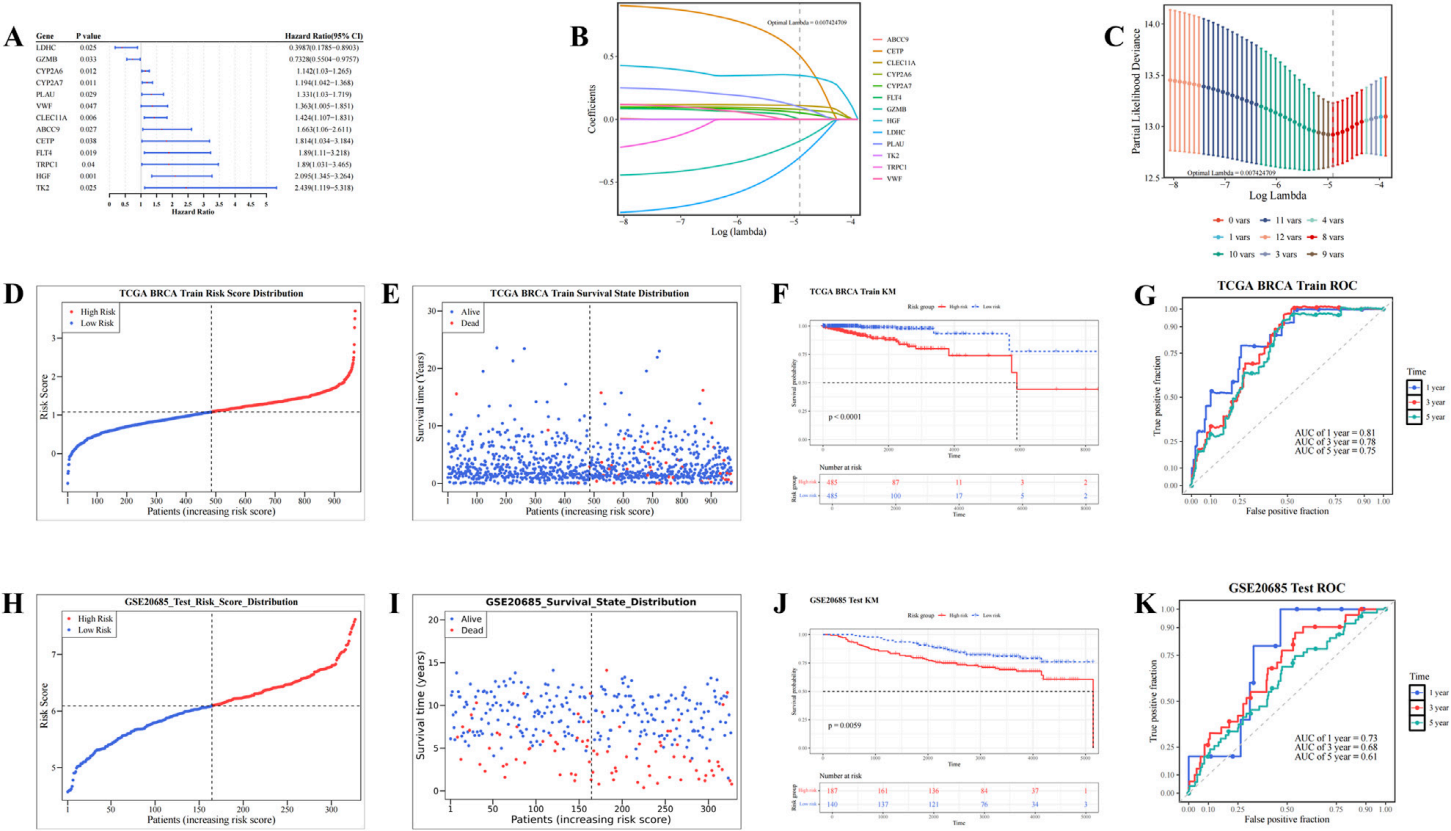
Screening of prognostic genes and construction of risk models. **(A)** Forest plot of univariate Cox regression analysis for screening prognostic genes. **(B,C)** Construction of Least Absolute Shrinkage and Selection Operator (Lasso) model. **(D,E)** Risk curve and survival status distribution diagram (TCGA_BRCA dataset). **(F–G)** Kaplan-Meier (KM) survival curve and Receiver Operating Characteristic (ROC) curve (TCGA_BRCA dataset). **(H,I)** Risk curve and survival status distribution diagram (GSE20685 dataset). **(J,K)** KM survival curve and ROC curve (GSE20685 dataset).

In the TCGA-BRCA dataset, univariate Cox analysis identified 13 candidate prognostic genes based on the candidate genes (P < 0.05, HR≠1) and the PH test (P > 0.05). These genes included *ABCC9*, *CETP*, *CLEC11A*, *CYP2A6*, *CYP2A7*, *FLT4*, *GZMB*, *HGF*, *LDHC*, *PLAU*, *TK2*, *TRPC1*, and *VWF* (Figure 2A; Supplementary Table S5). LASSO analysis of these 13 genes (optimal lambda = 7.42 × 10^−3^) revealed eight prognostic genes: *CETP*, *CLEC11A*, *CYP2A6*, *CYP2A7*, *GZMB*, *HGF*, *LDHC*, and *PLAU* (Figures 2B,C). Based on the coefficients derived from LASSO analysis, a risk score for each patient with BC was calculated using the following formula: risk score = *CETP* × (5.08 × 10^−1^) + *CLEC11A* × (1.10 × 10^−1^) + CY*P*2A6 × (7.77 × 10^−2^) + *CYP2A7* × (5.14 × 10^−2^) + *GZMB* × (−1.74 × 10^−1^) + *HGF* × (3.49 × 10^−1^) + *LDHC* × (−3.03 × 10^−1^) + *PLAU* × (9.80 × 10^−2^).

Based on the optimal cutoff value of the risk scores (TCGA-BRCA dataset: 1.08033, GSE20685: 6.090217), patients with BC were stratified into HRG and LRG. As risk scores increased, the mortality rate among patients with BC also rose (Figures 2D,E). Furthermore, the HRG group exhibited significantly lower survival rates compared to the LRG group (P < 0.0001) (Figure 2F). The AUC values for the 1-, 3-, and 5-year ROC curves were 0.81, 0.78, and 0.75, respectively, all exceeding 0.6 (Figure 2G). These results demonstrate that the risk model provides accurate prognostic predictions for BC. Validation of the risk model in the GSE20685 dataset yielded results consistent with those from the TCGA-BRCA dataset (Figures 2H–K). These results confirm that the risk model developed in this study is reliable and effective for predicting the prognosis of patients with BC, offering a potential reference for the clinical development of personalized treatment strategies.

### 3.3 A nomogram model was constructed, and risk scores among clinical subtypes were compared

**FIGURE 3:**
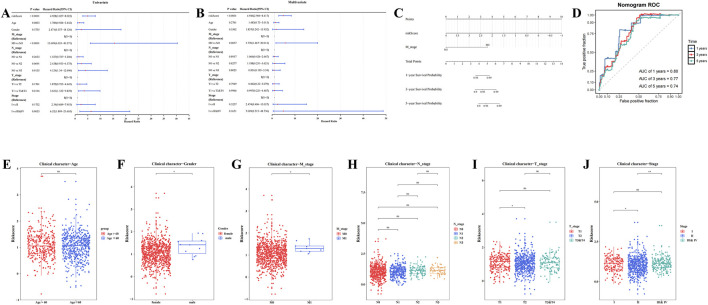
Construction of nomogram model and comparison of risk scores among clinical subtypes. **(A)** Forest plot of clinical features in univariate cox regression analysis. **(B)** Forest plot of clinical features in multivariate cox regression analysis. **(C)** Analysis of the nomogram model. **(D)** Validation of the nomogram model: Receiver Operating Characteristic (ROC) curve. **(E–J)** Analysis of risk score differences by clinical characteristics (**** represents p < 0.0001, *** represents p < 0.001, ** represents p < 0.01, * represents p < 0.05, ns represents not significant).

Risk score, T stage, N stage, M stage, and overall stage were identified as significant factors through univariate Cox analysis (HR≠1, P.adj <0.05) and the PH test (P > 0.05) (Figure 3A; Supplementary Table S6; Supplementary Figure S1). Specifically, M stage (P.adj <0.0001, HR = 4.946) and risk score (P.adj = 0.0057, HR = 5.77) were found to be independent prognostic factors following multivariate Cox analysis and the PH test (P > 0.05) (Figure 3B; Supplementary Table S7). These results indicate that M stage and risk score are independent prognostic variables that are strongly associated with the prognosis of patients with BC.

A nomogram model was then constructed based on the identified independent prognostic factors, M stage and risk score, allowing for the prediction of 1-, 3-, and 5-year survival probabilities for patients with BC. For each prognostic factor, a total score was calculated, with a higher score corresponding to a lower survival rate (Figure 3C). In ROC analysis, the AUC values for the 1-, 3-, and 5-year time points were 0.80, 0.77, and 0.74, respectively, all exceeding 0.6 (Figure 3D), demonstrating that the nomogram model possesses a high degree of predictive accuracy.

Significant variations in risk scores were observed across different clinical subgroups, including gender, stage (II vs. III & IV), T stage (T1 vs. T2), M stage (M0 vs. M1), and stage (I vs. II) (P.adj <0.05) (Figures 3E–J). The results of internal validation using Bootstrap (Supplementary Figure S2) showed that the AUC values of the model at the 1-year, 3-year and 5-year time nodes were all greater than 0.6, indicating that the model has good predictive efficacy in multiple time dimensions and is able to stably differentiate between high-risk and low-risk patient groups. These results suggest that clinical characteristics play a pivotal role in risk assessment and may guide the development of personalized treatment strategies for patients with BC.

### 3.4 GSVA within the HRG and LRG and GSEA of prognostic genes

**FIGURE 4:**
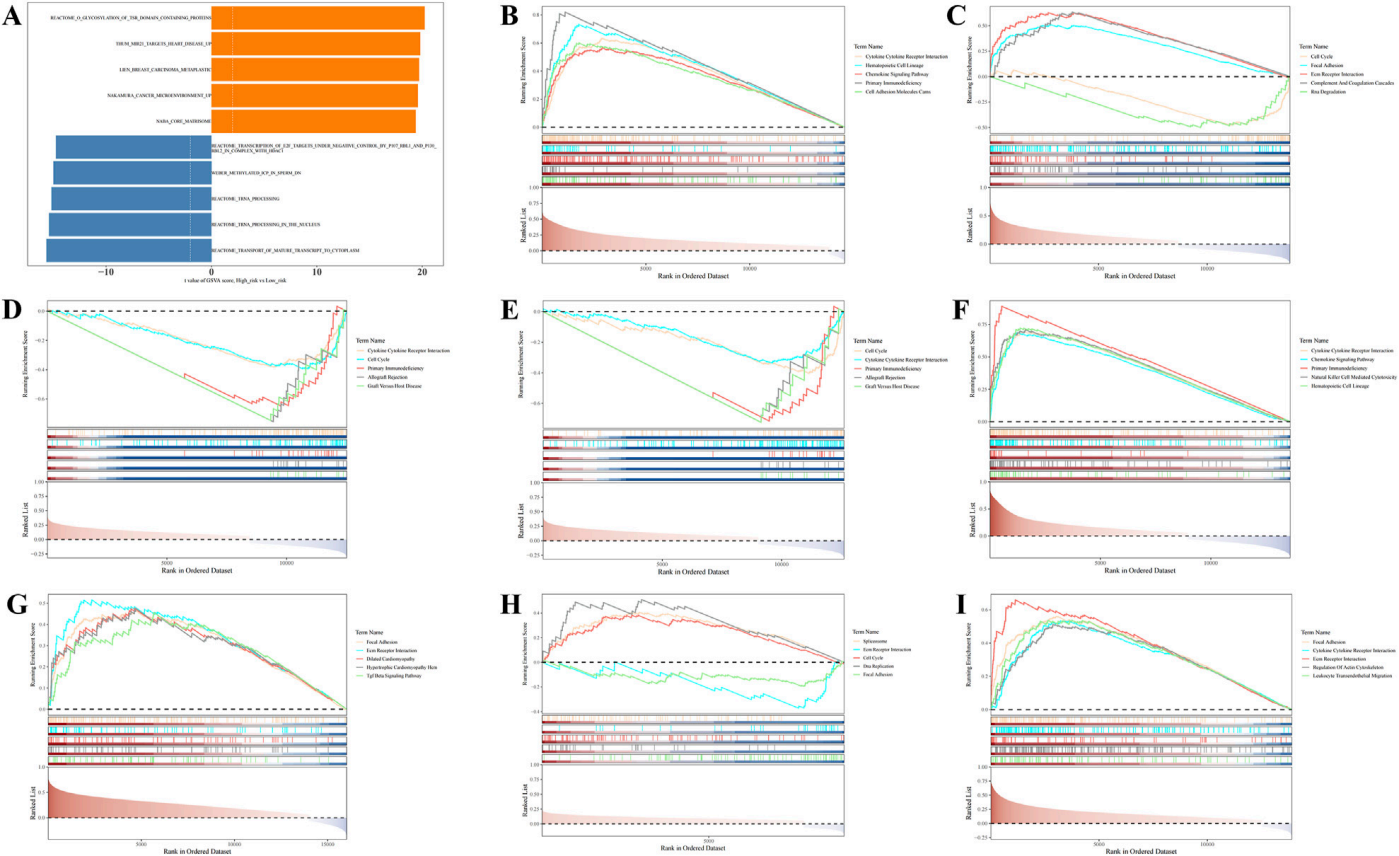
Gene Set Variation Analysis (GSVA) and Gene Set Enrichment Analysis (GSEA) enrichment analysis of prognostic genes. **(A)** GSVA enrichment analysis. **(B–I)** GSEA enrichment analysis.

GSVA revealed significant differences between the HRG and LRG groups in 5,510 KEGG pathways, such as the transport of mature transcripts to the cytoplasm and tRNA processing in the nucleus (P.adj <0.05) (Figure 4A; Supplementary Table S8). These pathways are likely to play critical roles in risk stratification for BC, providing valuable insights for further investigation into the disease’s pathogenesis and the development of targeted therapies.

Additionally, GSEA revealed that, except for *LDHC*, the remaining seven prognostic genes were significantly enriched in the cytokine-cytokine receptor interaction pathway (Figures 4B–I; Supplementary Table S9). These results suggest that these genes are likely involved in intercellular signaling processes, playing a significant regulatory role in the initiation, progression, and prognosis of BC.

### 3.5 Different immune microenvironments within HRG and LRG

**FIGURE 5:**
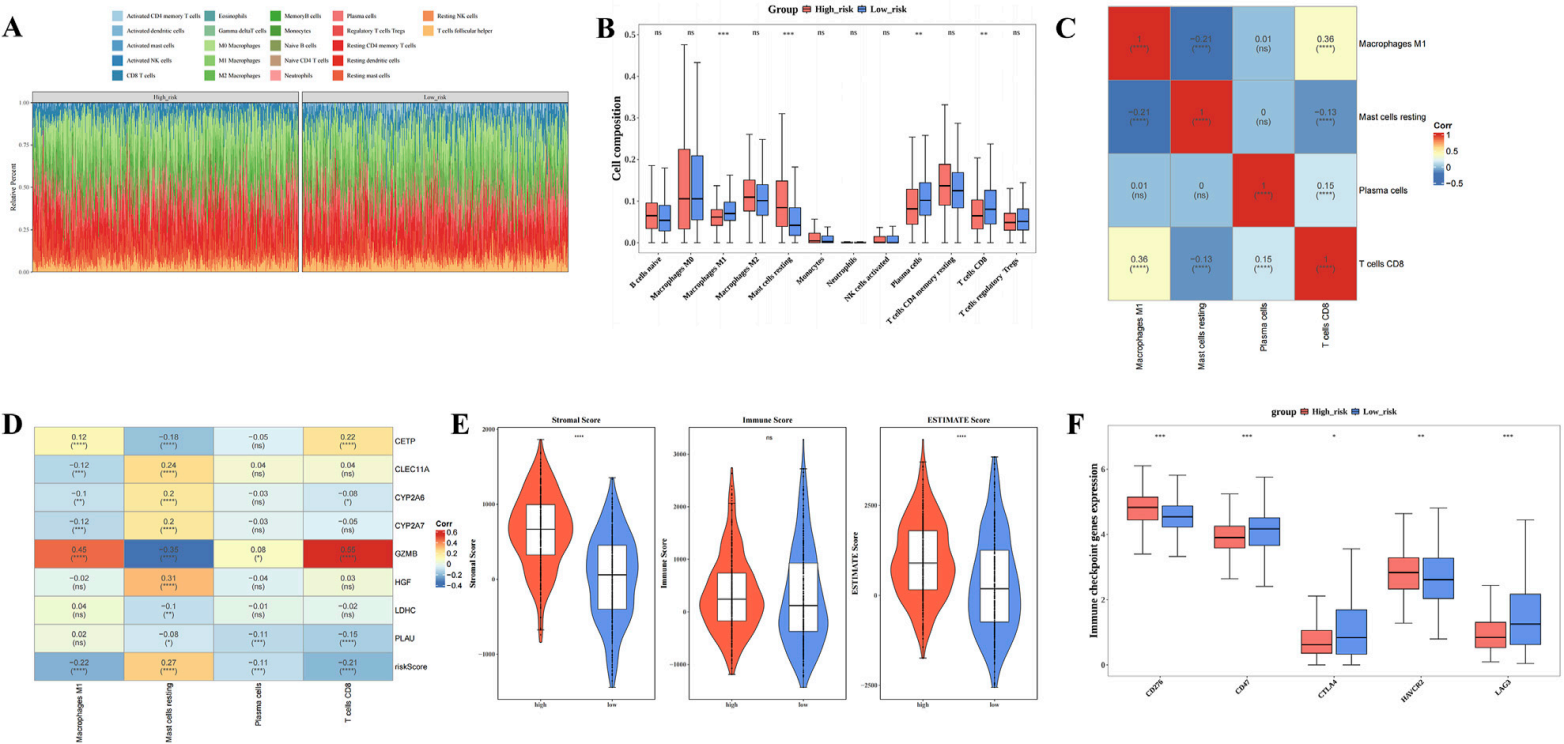
Different immune microenvironments within High risk group (HRG) and Low risk group (LRG). **(A)** Infiltration ratios of 22 distinct immune cell types within HRG and LRG. **(B)** Box plot of immune infiltrating cells based on the enrichment score between High/Low risk groups. **(C)** Correlation heat map between 4 types of immune cells. **(D)** Heat map of the correlation between 8 prognostic genes and 4 immune cells. **(E)** Differences in immune score, stromal score, and ESTIMATE score between low-risk and high-risk groups. **(F)** Box plot of differential expression of immune checkpoint genes between high-risk and low-risk groups. (**** represents p < 0.0001, *** represents p < 0.001, ** represents p < 0.01, * represents p < 0.05, ns represents not significant).

Figure 5A illustrates the infiltration levels of 22 distinct immune cell types in the HRG and LRG groups. There were 4 differentially abundant immune cell types within HRG and LRG, namely plasma cells, CD8 T cells, M1 macrophages, and resting mast cells (P.adj <0.05) Among them, resting mast cells demonstrated elevated infiltration extents in HRG, while the other three differentially abundant immune cell types displayed elevated infiltration degrees in LRG (Figure 5B). Furthermore, CD8 T cells demonstrated the strongest correlation with M1 macrophages (cor = 0.36, P.adj <0.05) (Figure 5C). *GZMB* exhibited a strong positive correlation with CD8 T cells (cor = 0.55, P.adj <0.05) but a significant negative correlation with resting mast cells (cor = −0.35, P.adj <0.05) (Figure 5D). These results suggest that the differing immune cell infiltration patterns and their associations with prognostic genes like *GZMB* contribute to the distinct immune microenvironments in HRG and LRG, may offer new insights into the immunomodulatory mechanisms involved in BC progression and provide guidance for the development of targeted immunotherapies. In addition, both stromal and ESTIMATE scores were significantly higher in the HRG group compared to the LRG group (P < 0.0001) (Figure 5E). Analysis of differential immune checkpoints within the two risk groups disclosed that 5 immune checkpoints, namely CD276, HAVCR2, CD47, CTLA4, LAG3, exhibited marked disparities within two risk groups (P.adj <0.05). Among them, CD276 and HAVCR2 were prominently expressed in HRG, while CD47, CTLA4, LAG3 were markedly expressed in LRG (Figure 5F). These differential immune checkpoint expressions not only reflect distinct immune evasion mechanisms in patients with BC exhibiting different risk profiles but also highlight critical targets for the design of personalized immunotherapy strategies.

### 3.6 Different genetic mutations within HRG and LRG

**FIGURE 6:**
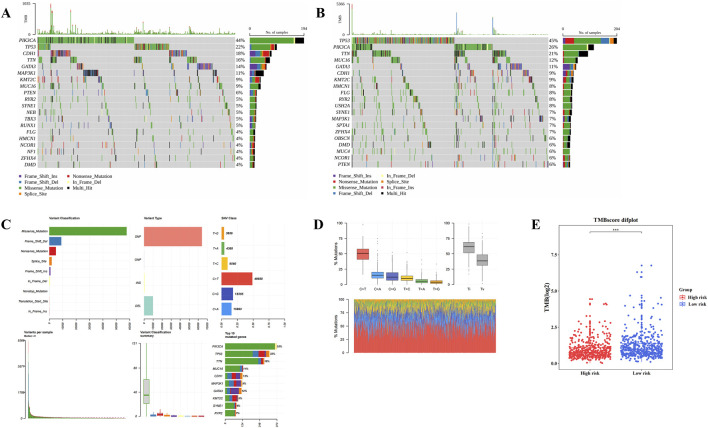
Different genetic mutations within High risk group (HRG) and Low risk group (LRG). **(A,B)** The top 20 genes with the highest mutation frequency between the high-risk group and the low-risk group. **(C,D)** The most common base substitution observed in Breast cancer (BC) samples was Cytosine (C) > Thymine (T). **(E)** Differences in tumor mutation burden (TMB) scores between high and low risk groups.

Gene mutation analysis revealed differences in mutation frequencies between the two risk groups. In both HRG and LRG, missense mutations were the most common type (Figures 6A,B), with C>T being the most frequent base substitution observed in BC samples (Figures 6C,D). Notably, the TMB was significantly higher in LRG compared to HRG (P < 0.001) (Figure 6E). These divergent gene mutation profiles between HRG and LRG suggest that patients with BC exhibiting different risk levels exhibit distinct tumor genomic stability, which is crucial for understanding BC heterogeneity and developing targeted treatment strategies.

### 3.7 Different drug sensitivities within HRG and LRG as well as TFs-mRNAs network of prognostic genes

**FIGURE 7:**
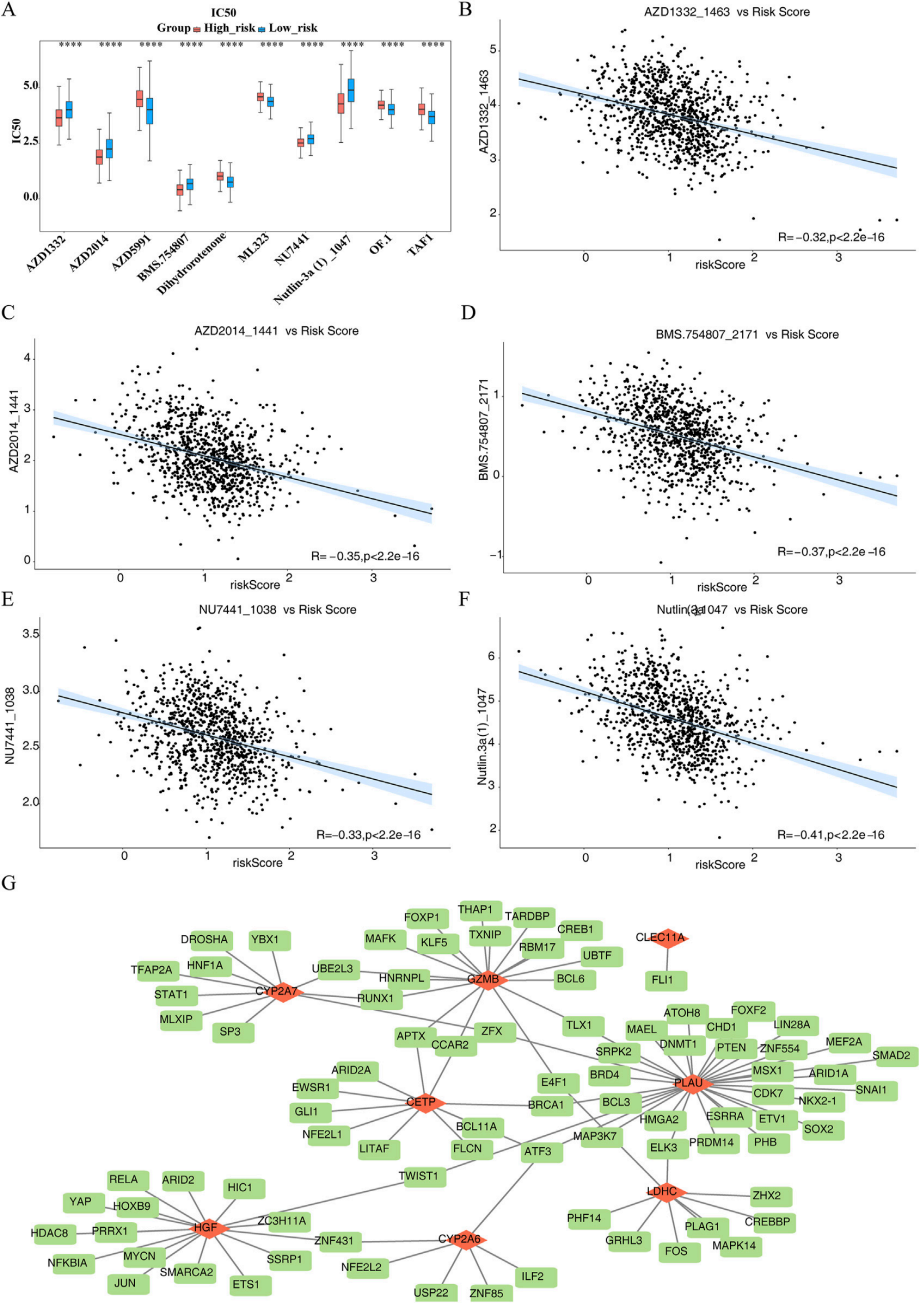
Different drug sensitivities within High risk group (HRG) and Low risk group (LRG) as well as Transcription Factors-messenger RNAs (TFs-mRNAs) network of prognostic genes. **(A)** Box plot of drug sensitivity prediction between high and low risk groups. **(B–F)** 5 drugs demonstrated a pronounced inverse association with risk score (P < 0.05, cor < −0.3). **(G)** Transcription Factors-messenger RNAs (TFs-mRNAs) network of prognostic genes.

The IC_50_ values of 133 drugs, including compounds like AZD1332, AZD2014, AZD5991, and BMS-754807, displayed significant variations between the HRG and LRG groups (Supplementary Table S10). The top 10 drugs with the most notable differences in IC_50_ values are shown in Figure 7A. AZD1332 and AZD2014 exhibited significantly higher IC_50_ values in the LRG group compared to HRG (P.adj <0.001), suggesting reduced effectiveness in LRG. Among the top 10 drugs with significant differences, AZD1332, AZD2014, BMS-754807, NU7441, and Nutlin.3a demonstrated a strong negative correlation with the risk score (P < 0.05, cor < −0.3) (Figures 7B–F). In contrast, the remaining five drugs showed a significant positive correlation with the risk score (P.adj <0.05, cor >0.3) (Supplementary Figure S3).

TF prediction for the regulation of prognostic genes revealed that *CETP*, *CLEC11A*, *CYP2A6*, *CYP2A7*, *GZMB*, *HGF*, *LDHC*, and *PLAU* were associated with 9, 1, 6, 10, 17, 16, 9, and 32 TFs, respectively. Notably, ATF3 was identified as a common TF for *CETP*, *PLAU*, and *CYP2A6* (Figure 7G). This suggests that ATF3 may play a critical regulatory role in modulating the expression of these three genes, potentially influencing related physiological and pathological processes.

### 3.8 The prognostic genes exhibited distinct content patterns in BC tumor tissues compared to normal tissues

**FIGURE 8:**
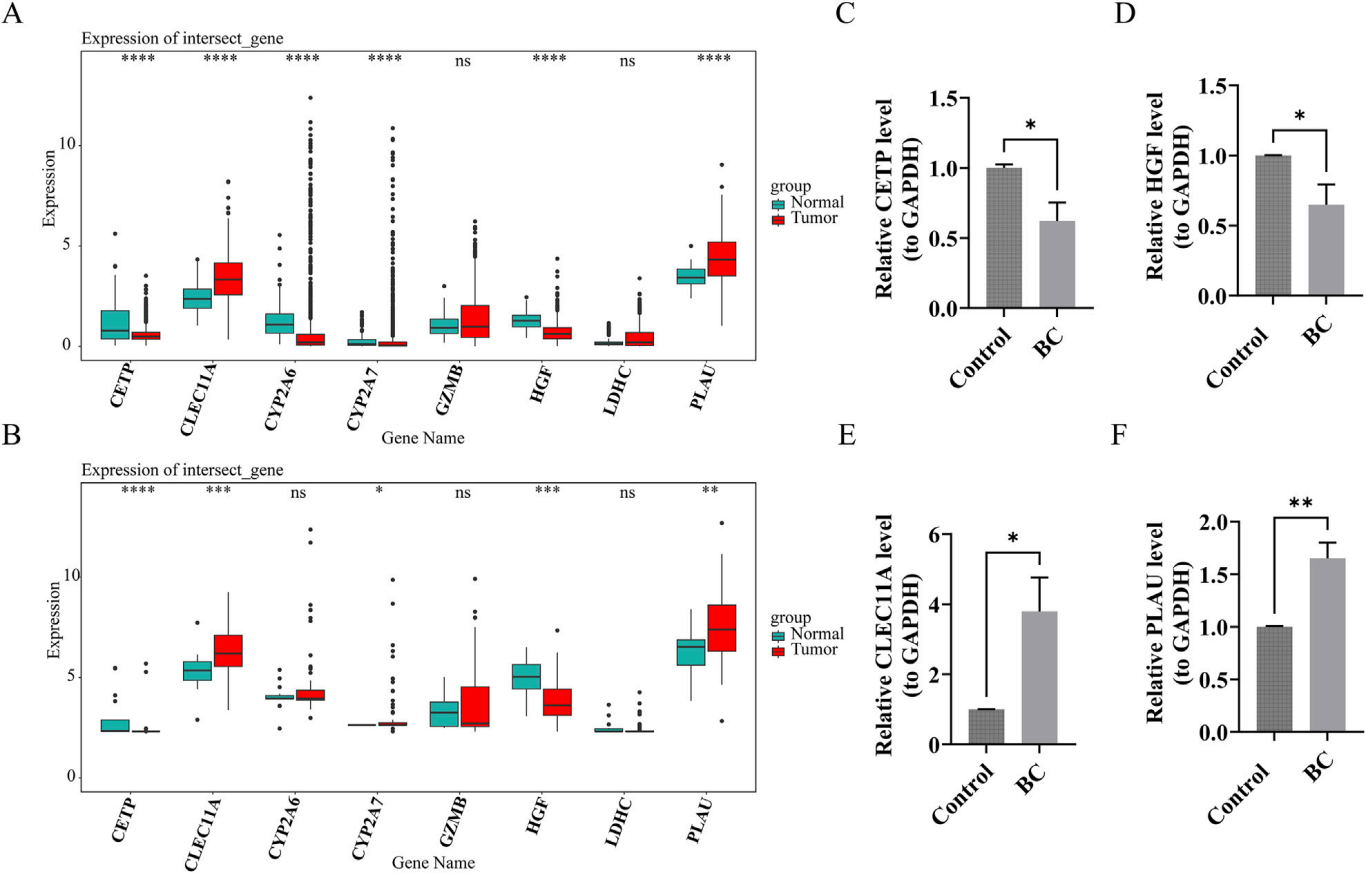
The prognostic genes exhibited distinct content patterns in Breast cancer (BC) tumor tissues compared to normal tissues. **(A,B)** Expression of prognostic genes in BC group and control group (TCGA-BRCA and GSE42568 datasets). ns indicates p > 0.05, * indicates p < 0.05, ** indicates p < 0.01, ***indicates p < 0.001, **** indicates p < 0.0001. **(C–F)** Verification of prognostic genes by reverse transcription-quantitative polymerase chain reaction (RT-qPCR) experiments. * indicates p < 0.05, ** indicates p < 0.01.

In both the TCGA-BRCA and GSE42568 datasets, *CETP*, *CLEC11A*, *HGF*, and *PLAU* exhibited significant differential expression between BC and normal tissue samples (P.adj <0.05). In normal samples, *CETP* and *HGF* had higher expression levels, while *CLEC11A* and *PLAU* were more abundant in BC samples (Figures 8A,B). These findings were consistent with the results from RT-qPCR experiments (Figures 8C–F), confirming the reliability of the bioinformatics analysis outcomes.

## 4 Discussion

BC is the most prevalent cancer among women. According to the 2018 Globocan report, it accounts for 6.6% of all cancer-related deaths globally (Freddie Bray et al., 2018), representing a major health challenge. Thus, identifying more effective treatment strategies is urgently needed. The redox state of Cyt c influences the resistance of BC cells to anti-cancer therapies (Barros et al., 2013). In caspase-9/Cyt c-mediated apoptosis, certain compounds have demonstrated the ability to inhibit the growth of triple-negative BC (TNBC) cells (Zhao et al., 2017). The mitochondrial apoptosis pathway, which is triggered by the release of Cyt c from the mitochondria, may be pivotal in inducing apoptosis in BC cells, thus playing a critical role in the treatment and prevention of BC (Lu et al., 2015; Hafezi et al., 2020). Although a strong association between Cyt c and BC has been established, the precise mechanisms of its action remain unclear. This study identified eight prognostic genes (*CETP*, *CLEC11A*, *CYP2A6*, *CYP2A7*, *GZMB*, *HGF*, *LDHC*, and *PLAU*) that significantly contribute to the onset and progression of BC, potentially offering new avenues for therapeutic intervention.

Candidate gene enrichment analysis showed that cytokine activity, PI3K-Akt signaling pathway, etc. are closely related to breast cancer. Specifically, cytokines, as key regulatory factors of the immune system, play a complex role in the breast cancer microenvironment (Shukla et al., 2024). For example, IL-6 is highly expressed in this microenvironment and can activate the STAT3 pathway through paracrine and autocrine pathways, thereby promoting breast cancer cell proliferation (Sun et al., 2019). The PI3K-Akt signaling pathway plays an important role in a variety of biological behaviors such as cell proliferation, apoptosis, invasion, migration, and glucose metabolism (Su et al., 2024). Its inhibition can enhance the efficacy of endocrine therapy for ER-positive breast cancer (Ciruelos Gil, 2014). Abnormal changes in this pathway are one of the main factors driving the growth, survival, and movement of breast cancer cells, and are also an important target for therapeutic intervention (Nunnery and Mayer, 2020). These results suggest that cytokine activity and the PI3K-Akt signaling pathway may be involved in the occurrence and development of breast cancer through multiple mechanisms, providing a direction for a deeper understanding of the disease mechanism and the development of targeted treatment strategies.

During the development and progression of breast cancer, a variety of molecules play important roles through different biological mechanisms. In terms of immune regulation, *GZMB* (granzyme B) is a serine protease that plays a key role in the immune system, especially in cell-mediated apoptosis. It is secreted by cytotoxic T lymphocytes (CTLs) and natural killer cells (NK cells) and can target and destroy infected or malignant cells (Lu et al., 2024; Liang et al., 2022; Li Z et al., 2024). Studies have shown that *GZMB* is a potential prognostic marker for colorectal cancer (Noti et al., 2022) and is involved in the progression of liver cancer (Gao et al., 2023). In breast cancer, its low expression is associated with poor survival outcomes, making it a practical prognostic biomarker for patients with PD-L1-positive triple-negative breast cancer (Zhong et al., 2022). In addition, the role of *GZMB* in the tumor microenvironment is of great significance for breast cancer immunotherapy (Larimer et al., 2017). In triple-negative breast cancer (TNBC) tumors, when *GZMB* levels are high, the levels of tumor-infiltrating lymphocytes are also high (Han et al., 2024).

In terms of metabolic regulation, cholesterol ester transfer protein (*CETP*) is a plasma glycoprotein synthesized by the liver. Its main function is to mediate the transfer of cholesterol esters from high-density lipoprotein (HDL) to lipoproteins containing apolipoprotein B (apoB) (such as low-density lipoprotein LDL and very low-density lipoprotein VLDL) and promote triglyceride exchange between different lipoproteins. At the same time, it is also involved in the reverse cholesterol transport system and plays a key role in maintaining lipid metabolism balance in the body (Aguchem et al., 2024; Schmidt et al., 2021; Blauw et al., 2020). Given that cholesterol can accelerate the progression of ER-positive breast cancer (Gu et al., 2024), understanding cholesterol homeostasis is crucial for breast cancer treatment. *CETP* plays a core role in maintaining the balance of cholesterol inside and outside the cell. Its function is closely related to the aggressiveness of breast cancer and is a potential therapeutic target with pharmacological significance. However, the molecular mechanism behind this association has not yet been clarified (Esau et al., 2016). The lactate dehydrogenase C subunit encoded by the *LDHC* gene catalyzes the reversible conversion of lactate to pyruvate (Wang et al., 2021). Its expression can promote tumor cell migration and invasion and the growth and metastasis of xenograft tumors. Specifically, it regulates cell proliferation and epithelial-mesenchymal transition by activating the PI3K/Akt/GSK-3β signaling pathway (Chen et al., 2021). The *LDHC*-STAT3 signaling axis plays a key role in regulating breast tumor cell survival (Naik et al., 2024). In breast cancer cell lines, silencing *LDHC* leads to nuclear abnormalities, DNA damage, and increased apoptosis (Naik and Decock, 2022). Cytochrome P450 superfamily members *CYP2A6* and *CYP2A7* are involved in the metabolism of a variety of endogenous and exogenous compounds, and the metabolites they produce play a key role in the carcinogenic process. Studies have found that genetic polymorphisms of cytochrome P450 enzymes are significantly associated with survival outcomes in breast cancer patients receiving adjuvant tamoxifen therapy (Chan et al., 2022). *CYP2A6* polymorphisms are also associated with a variety of cancers, including lung cancer, gastric cancer, and bladder cancer (Ezzeldin et al., 2018; Abudushataer et al., 2020; Kumondai et al., 2016), and the aromatase inhibitor letrozole is metabolized by *CYP2A6* and is commonly used to treat hormone receptor-positive early breast cancer (Puszkiel et al., 2024). In addition, *CYP2A7* is involved in hormone-related metabolic pathways and is closely related to the prognosis of patients with triple-negative breast cancer (Chen et al., 2020). However, more research is needed to better understand the molecular roles of *CYP2A6* and *CYP2A7* in breast cancer.

In terms of signaling pathways and tumor progression, hepatocyte growth factor (*HGF*) is a multifunctional growth factor that participates in a variety of physiological and pathological processes. It is mainly secreted by fibroblasts and exerts its effects by binding to the receptor c-MET. After *HGF* activates c-MET, it triggers a series of carcinogenic processes, including cell proliferation, metastasis, angiogenesis, and immunosuppression (Jabbarzadeh Kaboli et al., 2024). Among them, the *HGF*/c-MET pathway is closely related to cancer metastasis and can promote breast cancer resistance to tamoxifen through the EZH2/HOTAIR-MIR-141/200A feedback signaling pathway (Lai et al., 2025). *HGF* is a key pathway in the occurrence of breast cancer and has racial expression differences (Jones et al., 2022). There are significant differences in serum *HGF* levels between breast cancer patients and healthy people, especially in postmenopausal women, poorly differentiated tumors, and distant metastatic breast cancer patients. At the same time, its expression is strongly correlated with mitotic counts and nuclear polymorphisms (Pai and Kittur, 2023). The urokinase-type plasminogen activator (uPA) encoded by the *PLAU* gene is a serine protease that plays a key role in processes such as extracellular matrix degradation and cell migration (Shi et al., 2024). Studies have shown that this gene is involved in regulating a variety of cancers, including Wilms tumor, lung cancer, and pancreatic cancer (Li D et al., 2024; Guo et al., 2024; Hosen et al., 2022). In breast cancer, elevated *PLAU* expression levels are closely associated with prognosis, invasiveness, metastasis, and tumor infiltration (Gouri ADKE, 2016; Duffy et al., 2014). As a gene associated with aging, it is not only related to immune cell infiltration in breast cancer, but also closely related to resistance to chemotherapy and targeted therapy (Li et al., 2023).

In addition, *CLEC11A* (also known as stem cell growth factor SCGF or myeloid lectin) belongs to the C-type lectin superfamily. It was originally identified as an autocrine factor secreted by leukemia cell lines and plays an important role in angiogenesis. Its expression level is often increased in lung adenocarcinoma tissues with epidermal growth factor receptor (EGFR) mutations (Lin et al., 2022). In gastric cancer, overexpression of *CLEC11A* is associated with poor prognosis (Zheng et al., 2024). In addition, it is also associated with acute myeloid leukemia, head and neck squamous cell carcinoma, pancreatic cancer and other cancers (Yin et al., 2021; Wu et al., 2022; Kisi et al., 2015). Although the current research on *CLEC11A* in breast cancer is limited, it may become a potential prognostic and immune biomarker for the disease.

The direct interaction between Cyt c and *CETP*, *CLEC11A*, *CYP2A6* and *CYP2A7* has not been clearly revealed, but they have potential indirect associations in cholesterol metabolism, mitochondrial function, oxidative stress regulation and tumor microenvironment regulation. Studies have shown that *CETP* in cancer cells may affect the release of Cyt c and apoptosis sensitivity by changing mitochondrial cholesterol metabolism (Pessoa, 2022). *CLEC11A* may reduce the level of intracellular reactive oxygen species (ROS) by enhancing the activity of antioxidant enzymes (such as SOD, CAT), thereby reducing oxidative stress-induced Cyt c release (Guerra-Castellano et al., 2018; Srinivasan and Avadhani, 2012). *CYP2A6* generates ROS during liver metabolism (Tanner and Tyndale, 2017), the accumulation of ROS leads to lipid peroxidation of mitochondrial membranes and damages mitochondrial membrane potential, which in turn promotes the release of Cyt c from the inner mitochondrial membrane into the cytoplasm, activates apoptotic proteases, and initiates the apoptotic cascade (Reshi et al., 2017). *CYP2A7* competitively binds to miR-126, relieves its inhibitory effect on *CYP2A6*, and indirectly increases ROS generation and Cyt c release (Nakano et al., 2015). Cyt c and *GZMB* have a clear functional association in the regulation of apoptosis, mainly through the mitochondria-dependent apoptosis pathway. Studies have shown that in the breast cancer cell line MCF-7, *GZMB* synergizes with perforin to induce Bid cleavage and Bax/Bak oligomerization, significantly promoting Cyt c release and caspase-3 activation (Afzal et al., 2024). As a mesenchymal marker in the tumor microenvironment, *HGF*’s high expression is associated with reduced Cyt c release and caspase-3 activity in TNBC, suggesting that inhibition of the mitochondrial apoptosis pathway is an important mechanism for *HGF* to promote cancer (Jones et al., 2021). In the triple-negative breast cancer MDA-MB-231 cell line, overexpression of *LDHC* inhibits the function of mitochondrial respiratory chain complexes and reduces mitochondrial outer membrane permeability (MOMP) to limit the release of Cyt c, thereby inhibiting the apoptosis process (Kong et al., 2016). Studies have shown that *PLAU* inhibits Cyt c release by activating the PI3K/AKT and NF-κB signaling pathways, and its high expression is associated with poor prognosis in breast cancer patients. Targeting *PLAU* may enhance the effect of chemotherapy by restoring the pro-apoptotic function of Cyt c (Mahmood et al., 2018). In summary, these associations suggest that Cyt c can serve as a potential important node for evaluating the prognosis of breast cancer, and prognostic genes can provide a new approach for the treatment of breast cancer by monitoring their regulatory effects on Cyt c.

In breast cancer, transport of mature transcripts to the cytoplasm, tRNA processing in the nucleus and cytokine-cytokine receptor interaction pathway directly or indirectly regulate the localization, function and mitochondrial release of Cyt c, thereby affecting the apoptotic balance and malignant progression of tumor cells. The nuclear-cytoplasmic transport of mature transcripts depends on the nuclear pore complex and transport receptors such as XPO1 and CRM1. In triple-negative breast cancer, high expression of XPO1 accelerates proliferation by exporting oncogene mRNAs such as MYC and ERBB2, inhibits mitochondrial release of Cyt c, and weakens apoptotic sensitivity (Zhao et al., 2021). The interaction between tRNA processing in the nucleus and Cyt c is a key link in affecting breast cancer cell apoptosis. tRNA can inhibit the formation of apoptotic bodies and the activation of caspase-9 by blocking the binding of Cyt c to Apaf-1, and reduce the peroxidase activity of Cyt c (related to mitochondrial release), thereby doubly inhibiting its pro-apoptotic function (Mei et al., 2010; Liu et al., 2016). Regarding cytokine-cytokine receptor interaction pathway, the study found that in triple-negative breast cancer, the IL-6 autocrine loop can also enhance EMT-related gene transcription and improve cell migration ability, and its mechanism may be related to the continuous inhibition of Cyt c-mediated apoptosis (Autenshlyus et al., 2021). VEGF-B reduces Cyt c release by maintaining mitochondrial membrane homeostasis and binding ability to cardiolipin (Cyt c’s mitochondrial inner membrane anchor) (He et al., 2024). In summary, targeting key molecules related to Cyt c function in these pathways (such as CRM1, tRNA binding sites, IL-6 receptors) may provide a new strategy for breast cancer treatment by restoring the pro-apoptotic activity of Cyt c.

In the immune microenvironment analysis, we found that CD8^+^ T cells, M1 macrophages were more abundant in the low-risk group, and the infiltration of resting mast cells was significantly increased in the HRG group. As core anti-tumor effector cells, CD8^+^ T cells inhibit Treg activity by secreting IFN-γ (Mortazavi Farsani et al., 2025; Li et al., 2010), and their infiltration is associated with good survival in patients with various cancers such as breast cancer (Nalio Ramos et al., 2022). *GZMB*, as a key effector molecule of cytotoxic immune cells, can induce apoptosis and pyroptosis of target cells through perforin-dependent or -independent mechanisms. Glucose deficiency and lactate accumulation in the tumor microenvironment inhibit CD8^+^ T cell glycolysis and reduce *GZMB* release (Thompson and Cao, 2024; Ringel et al., 2020). In addition, studies have shown that the polarization state of M1 macrophages can be regulated by cytokines and metabolic factors in the microenvironment, causing them to transform into M2 macrophages. This transformation process promotes tumor immune escape and progression (Bai et al., 2024), which in turn affects breast cancer. At the same time, resting mast cells can regulate the recruitment and activity of other immune cells by releasing cytokines and chemokines, thereby participating in the regulation of the immune balance of the tumor microenvironment (Gou et al., 2021). At the same time, mast cells can also release angiogenic factors such as vascular endothelial growth factor (VEGF) to promote tumor angiogenesis to provide nutritional support (Ribatti et al., 2021). These effects may affect the occurrence and development of breast cancer. In addition, the matrix and ESTIMATE scores can reflect the enrichment of stromal cells such as CAFs, and comprehensively evaluate the overall ratio of stromal cells to immune cells. The increase of both is closely related to the biological behavior of tumors. Among them, CAFs can promote tumor proliferation, migration and drug resistance by activating PI3K/AKT, STAT3 and other pathways through secreting CXCL12, IL-6, etc., and form a pro-cancer positive feedback loop with immunosuppressive cells such as MDSC and TAM (Wei et al., 2015; Guo et al., 2023; Tajaldini et al., 2022). In addition, the immune infiltration pattern affects disease progression through the dynamic balance between the adaptive immune response and the immunosuppressive network. Combining it with the risk score and other clinical characteristics can more accurately predict prognosis and treatment response (Xu et al., 2021). Studies have confirmed that CD8^+^ T cell infiltration is associated with a good prognosis in triple-negative breast cancer (Denkert et al., 2018; Loi et al., 2014), and high mRNA expression of GNLY and *GZMB* in tumors is associated with a good prognosis in colorectal cancer (Pages et al., 2005). These results support that CD8^+^ T cells, *GZMB*, etc. can be used as potential therapeutic targets, which is consistent with the objectives of this study.

In summary, this study successfully identified eight CCRGs (*CETP*, *CLEC11A*, *CYP2A6*, *CYP2A7*, *GZMB*, *HGF*, *LDHC*, and *PLAU*) that are closely associated with BC prognosis. These findings contribute to a deeper understanding of the molecular mechanisms and prognostic factors involved in BC development and provide a meaningful reference for studying the pathogenesis of BC and advancing clinical diagnosis and treatment. However, the study still has certain limitations. The platform differences and data quality fluctuations of different data sets may affect the consistency and repeatability of the results. Although this study uses a variety of correlation analysis methods, statistical correlation can only reflect the trend of synergistic changes between variables and cannot directly infer causal relationships. In addition, the hazard ratio of risk score to M stage in the report is relatively high, suggesting that the prognostic risk model may have the potential risk of overfitting. At the same time, the risk model of this study was established based on a public data set, and its clinical practice application needs further verification. Therefore, its results can only be used as a reference and have certain limitations in clinical practice application. In the future, we will systematically explore genes and enriched signaling pathways related to breast cancer prognosis, and verify their biological functions and associations with disease progression through functional experiments and mechanism studies. For the risk score and high risk ratio of M stage, we will combine statistical model optimization (such as regularization, cross-validation), multi-cohort data and biological mechanism analysis to systematically evaluate the authenticity of the results. At the same time, the risk model needs to be further verified through prospective cohort studies or functional experiments to better apply it to clinical practice. In addition, we will deepen the mechanism research and clinical application exploration of immune infiltration patterns, and through IHC verification of large sample cohorts, clarify the importance of prognostic genes in BC and protein expression levels, and provide new basis for accurate diagnosis, personalized treatment and prognosis evaluation of BC.

## 5 Conclusion

Based on publicly available BC transcriptome data, this study identified eight prognostic genes related to Cyt c in BC: *CETP*, *CLEC11A*, *CYP2A6*, *CYP2A7*, *GZMB*, *HGF*, *LDHC*, and *PLAU*. A risk model was developed, showing that high-risk patients with BC had significantly lower survival rates compared to low-risk patients. Independent prognostic analysis revealed that M stage and risk score were independent prognostic factors for BC. A nomogram model was effectively constructed based on these two factors. Furthermore, GSEA and TF-mRNA analysis were used to explore enriched pathways and TFs regulating the prognostic genes. The study also revealed differences in immune microenvironment, gene mutations, and drug sensitivity between high-risk and low-risk groups. In conclusion, this study identifies key prognostic genes related to Cyt c in BC, and through bioinformatics approaches, it explores the underlying mechanisms, offering new molecular targets and therapeutic strategies for the clinical treatment of BC.

## Data Availability

The datasets analyzed for this study can be found in the (GEO) (https://www.ncbi.nlm.nih.gov/geo/).
