# Supplementary material for: Identification and experimental validation of prognostic genes related to cytochrome c in breast cancer
Source: Front Genet. 2025 Aug 11;16:1627134. doi: 10.3389/fgene.2025.1627134 (PMC12375475; doi:10.3389/fgene.2025.1627134)
Supplement: Supplementary file 4 [file Table12.docx]

**Supplementary Figure S2**

**
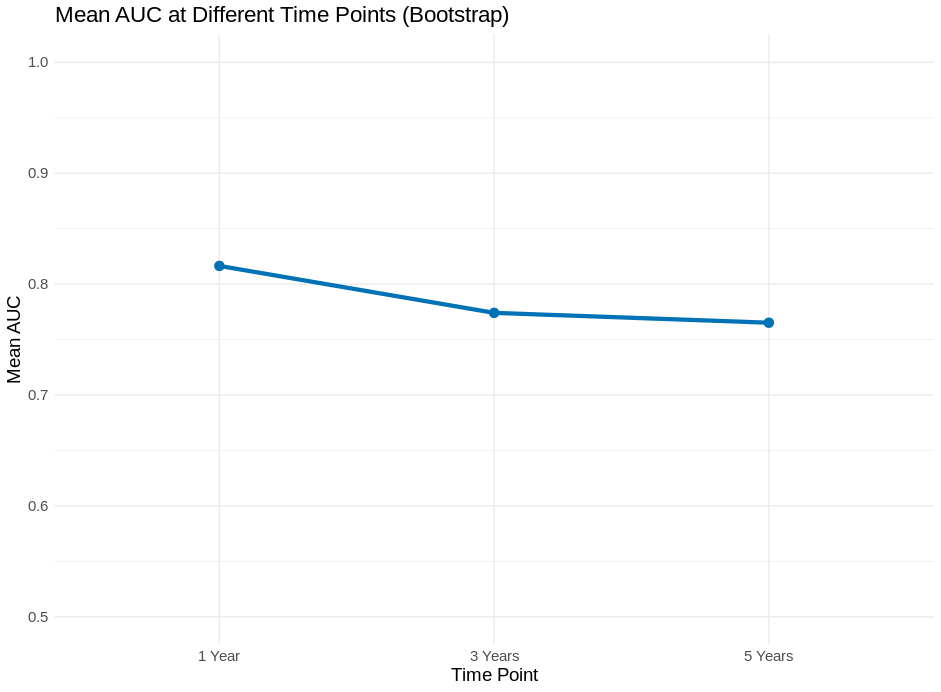
**

**The result diagram of the internal verification of the self-raise method.**


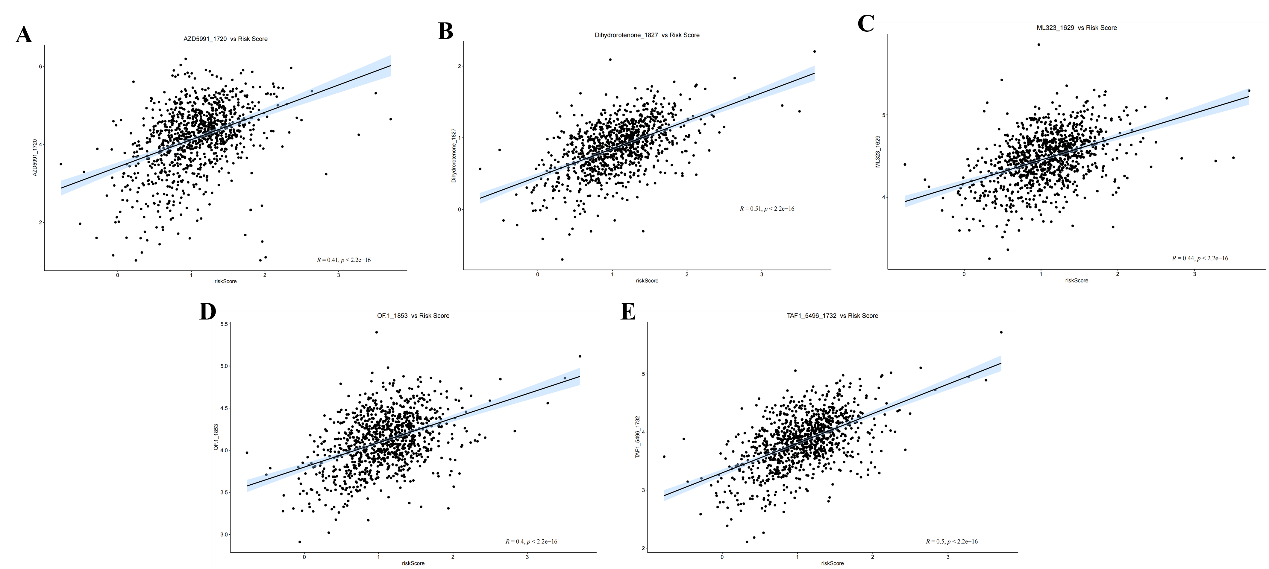


**Supplementary Figure S3** **Results of the correlation between drugs and risk scores**
