# Supplementary figures and images for: Identification and experimental validation of prognostic genes related to cytochrome c in breast cancer
Source: Front Genet. 2025 Aug 11;16:1627134. doi: 10.3389/fgene.2025.1627134 (PMC12375475; doi:10.3389/fgene.2025.1627134)

**Supplementary Figure S3**


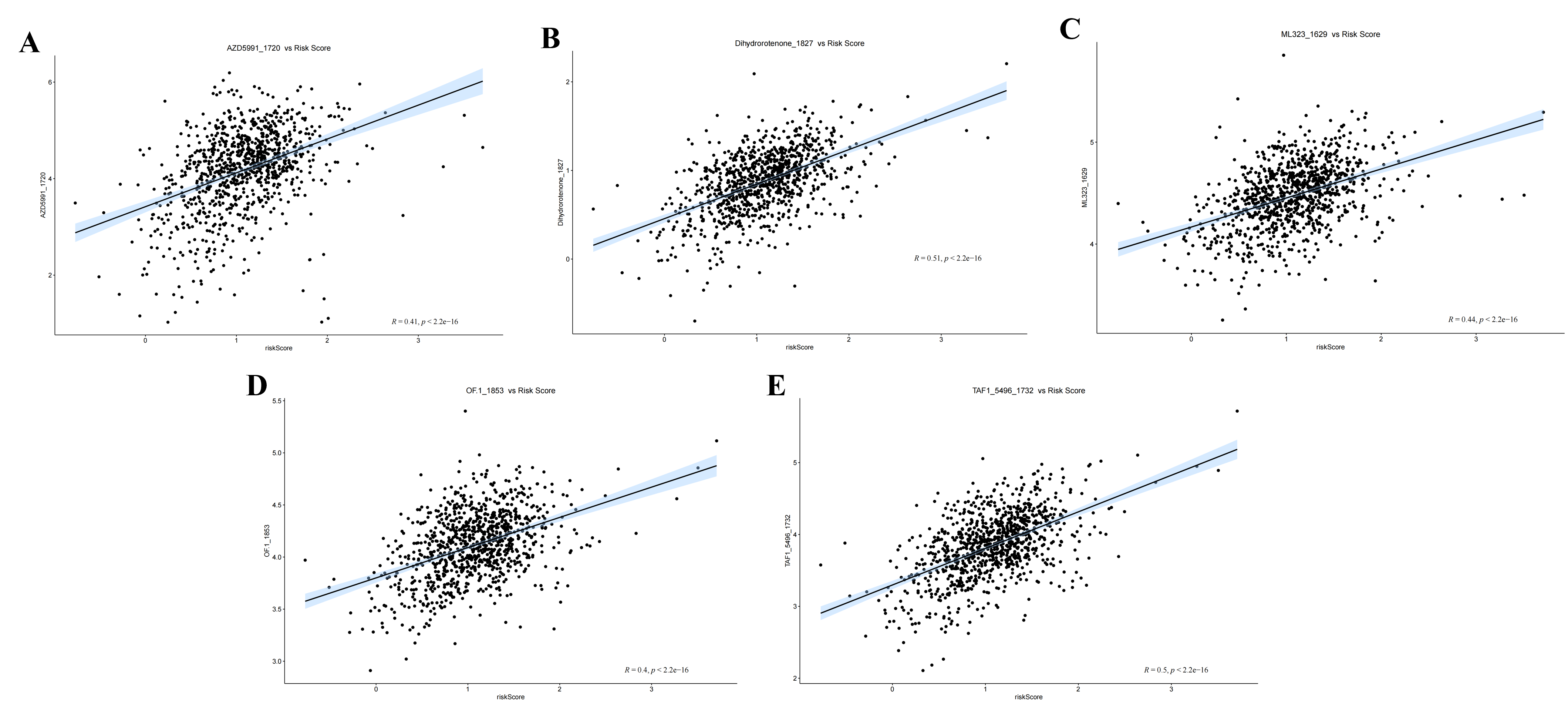


**Results of the correlation between drugs and risk scores**

Supplement: Supplementary file 7 [file Table13.docx]

**Supplementary Figure S1**


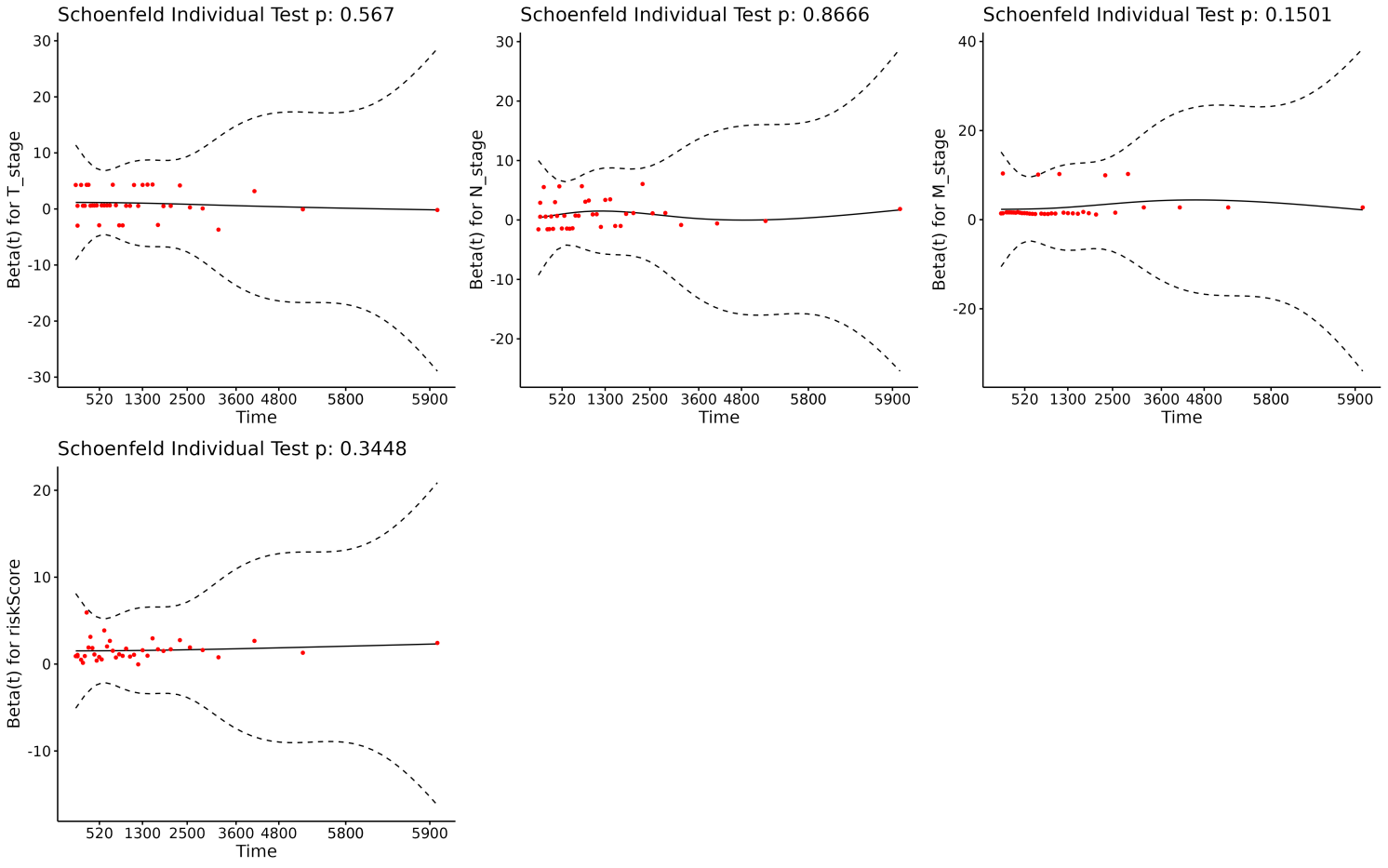


**Schoenfeld residual plot of the PH hypothesis test.**

Supplement: Supplementary file 9 [file Table11.docx]
